# An Adaptive Telephone Coaching Intervention for Patients in an Online Weight Loss Program: A Randomized Clinical Trial

**DOI:** 10.1001/jamanetworkopen.2024.14587

**Published:** 2024-06-07

**Authors:** Jessica L. Unick, Christine A. Pellegrini, Shira I. Dunsiger, Kathryn E. Demos, J. Graham Thomas, Dale S. Bond, Robert H. Lee, Jennifer Webster, Rena R. Wing

**Affiliations:** 1 Warren Alpert Medical School at Brown University, Providence, Rhode Island; 2 The Miriam Hospital Weight Control and Diabetes Research Center, Providence, Rhode Island; 3 Department of Exercise Science, Arnold School of Public Health, University of South Carolina, Columbia; 4 Department of Behavioral and Social Sciences, Brown University School of Public Health, Providence, Rhode Island; 5 Department of Surgery, Hartford Hospital, Hartford, Connecticut; 6 Department of Research, Hartford Hospital, Hartford, Connecticut; 7 School of Medicine, University of Kansas, Kansas City

## Abstract

**Importance:**

Weight loss (WL) during the first month of a behavioral program is associated with longer-term WL. Testing of translatable and adaptive obesity programs is needed.

**Objective:**

To compare brief, extended, and no telephone coaching for individuals with suboptimal response (ie, 1-month WL <4%) within an online WL program.

**Design, Setting, and Participants:**

This randomized clinical trial with enrollment between March 2019 and April 2022 (data collection completed May 2023) was conducted at an academic research center in the US. Eligible participants included adults aged 18 to 70 years with daily access to internet and a body mass index between 25 and 45.

**Interventions:**

All participants received an automated online WL program (4 months) and WL maintenance program (8 months), consisting of video lessons, self-monitoring, and personalized feedback. Participants were randomized, such that individuals with suboptimal response received either brief telephone coaching (3 calls during weeks 5-8), extended telephone coaching (12 calls during weeks 5-16), or no coaching (control). Coaching included education, problem solving, and goal setting, and promoted engagement with the online program.

**Main Outcomes and Measures:**

The primary outcomes were percent weight change and proportion of participants achieving 5% or greater WL at 4 and 12 months. A priori hypotheses for WL were that WL for extended coaching would be greater than for brief coaching, and both extended and brief coaching would be greater than no coaching (control). A longitudinal mixed-effects model with participant-specific intercept was used to examine intervention effects on percent WL at 4 and 12 months. Secondary analyses focused on program engagement and cost/kilogram of WL.

**Results:**

The study included a total of 437 participants who reported WL at 1 month (mean [SD] age, 50.8 [11.4] years; mean [SD] BMI, 34.6 [5.0]; 305 female [69.8%] and 132 male [30.2%]) with 148 randomized to extended coaching, 143 assigned to brief coaching, and 146 assigned to the control group. Of all participants, 346 (79.2%) were considered to have a suboptimal response. WL at 4 months was significantly greater in the extended coaching group (mean [SD] WL, −7.0% [5.1%]) and brief coaching group (mean [SD] WL, −6.2% [4.7%]) vs the control group (mean [SD] WL, −4.5% [4.7%]) (*P* < .001). Similarly, the proportion of participants achieving 5% or greater WL at 4 months was greater in the extended coaching group (89 participants [65.9%]) and brief coaching group (77 participants [58.5%]) vs control group (46 participants [36.5%]) (*P* < .001). At 12 months, a similar pattern was observed for achievement of 5% WL or greater (extended coaching, 63 participants [48.1%]; brief coaching, 58 participants [45.9%]; control, 38 participants [32.8%]; *P* = .03). Percent WL at 12 months was significantly higher in extended coaching vs control (mean [SD] WL for extended coaching, −5.5% [6.7%]; mean [SD] WL for control, −3.9% [7.4%]; *P* = .03) but not for brief coaching (mean [SD] WL, −4.9% [6.1%]).Both the brief and extended coaching groups watched more lessons and self-monitored on more days compared with the control group. The cost per additional kilogram of WL, beyond that of the control group, was $50.09 for brief coaching and $92.65 for extended coaching.

**Conclusions and Relevance:**

In this randomized clinical trial testing an adaptive intervention, the provision of coaching for individuals with suboptimal response improved WL and was cost-effective; further testing in clinical settings (eg, health care systems) is warranted.

**Trial Registration:**

ClinicalTrials.gov Identifier: NCT03867981

## Key Points

**Question:**

What is the effect of adding telephone coaching for individuals with suboptimal response enrolled in an online weight loss program?

**Findings:**

In this 3-group randomized clinical trial of 437 participants, the provision of 3 or 12 weeks of telephone coaching to those with suboptimal weight loss at 1-month improved weight loss outcomes and engagement with the online program compared with those not receiving coaching.

**Meaning:**

These findings suggest that adapting an online obesity treatment program by providing coaching to individuals with suboptimal response offers a cost-effective approach to remote obesity treatment; this treatment model should be further evaluated in other settings and patient populations.

## Teaser

This randomized clinical trial compares brief and extended coaching programs for patients in an online weight loss program who had achieved less than 4% of body weight loss at 1 month.

## Introduction

Effective weight loss (WL) interventions with high dissemination potential are important for addressing the obesity epidemic and obesity-related comorbidities. Currently, the US Preventive Services Task Force recommends referral of adults with obesity to intensive, multicomponent behavioral interventions, which include behavioral counseling and lifestyle modification for diet and exercise.^1^ While intensive behavioral programs can produce clinically significant WL and improvements in cardiometabolic risk factors,^2,3^ they are limited in reach due to geographical constraints, availability of programs, cost, and time-related barriers. Conversely, fully automated online behavioral WL programs can reduce costs and reach more individuals; however, WL produced via these programs is less than traditional behavioral therapy, and large variability is observed.^4,5^

One potential strategy to improve the effectiveness of online WL programs is via an adaptive intervention approach.^6,7^ Early WL (eg, 1 month) is associated with longer-term WL^8,9,10,11^; thus, identifying and providing additional intervention to individuals with suboptimal response could enhance the effectiveness of online programs. This represents a cost-effective and translatable WL treatment model where patients are referred to a low-cost, low-intensity intervention (eg, a fully automated online program), and additional, more costly and intensive personalized treatment is reserved for those who fail to achieve early WL goals.

To date, few studies have tested rescue approaches for individuals with suboptimal response.^12,13^ One study^13^ found that 3 weeks of coaching for individuals with nonresponse (ie, 1-month WL<2.3%) of an automated online program improved program adherence and nearly doubled 12-week WL compared with individuals with nonresponse who did not receive coaching. However, the sample size was limited, participants were not followed-up beyond 12 weeks, and it is uncertain whether a longer period of coaching would have produced even greater benefits.

This randomized clinical trial was designed to compare 2 doses of telephone coaching (brief, inlcluding 3 telephone calls; extended, including 12 telephone calls) for responders with subuptimal WL response in an online WL program, relative to a no-coaching control condition. The primary aims were to compare treatment groups, which included all randomized participants, on percent weight change from baseline to 4- and 12-month follow-up and to assess the proportion of participants in each group achieving clinically significant WL (ie, ≥5%). It was hypothesized that WL would be greater in both brief and extended coaching relative to control and that extended coaching would produce greater WL than brief coaching. Secondary aims compared treatment groups on the cost per kilogram of WL and program engagement (self-monitoring and video lessons viewed), assessed weight outcomes among individuals with suboptimal response only (vs all randomized participants), and examined whether 1-month WL was a moderator of the treatment effect.

## Methods

### Study Design

This study was approved by the Miriam Hospital institutional review board and followed the Consolidated Standards of Reporting Trials (CONSORT) reporting guideline. The Disseminating Internet-Based Approaches for Lifelong Change (DIAL) Now Trial^14^ was a 3-group randomized clinical trial designed to test the efficacy of adding brief or extended phone coaching to an online WL program for individuals with suboptimal response (ie, 1-month WL<4%). This single-site trial was conducted at Miriam Hospital in Providence Rhode Island and enrollment occurred between March 6, 2019, and April 27, 2022, with final data collection completed in May 2023. All participants provided written informed consent. A detailed description of the study design and methods has been reported previously^14^ and the trial protocol is available in Supplement 1.

### Participants

A total of 452 WL–seeking adults aged 18 to 70 years with daily internet access and a body mass index (BMI) of 25 to 45 (calculated as weight in kilograms divided by height in meters squared) enrolled in this trial. Exclusion criteria included current or recent enrollment in another WL program, pregnancy, history of bariatric surgery, WL medication use, inability to walk several blocks unassisted, plans to relocate outside the geographic area within 12 months, or presence of any medical condition for which WL was contraindicated.

### Recruitment and Screening

Participants were recruited via social media platforms and direct mailings, targeting residents of Rhode Island and southeastern Massachusetts. Interested individuals completed an eligibility screener via a universal recruitment website, and those deemed initially eligible were contacted by research staff for a telephone screen. Eligible candidates were scheduled for an orientation and consenting session and baseline appointment. Completion of all baseline procedures was required for randomization.

### Randomization

Participants were randomized at baseline to receive online WL treatment and (1) brief telephone coaching for individuals with suboptimal response (ie, the brief group), (2) extended telephone coaching for individuals with suboptimal response (ie, the extended group), or (3) no additional support (ie, the control group). Participants were not informed of their randomization assignment until month 1 into the study and they were never informed of the criteria for determining who would receive coaching. Randomization by the study biostatistician (S.D.) occurred in equal numbers based on a permutated block randomization procedure with small, random-sized blocks. Randomization was stratified by sex assigned at birth (male or female), baseline BMI (<35 or ≥35), and self-reported race and ethnicity (non-Hispanic White individuals and those from an underrepresented racial or ethnic group) to ensure equal representation across intervention groups. Ethnicity categories included Hispanic and non-Hispanic. Race categories included African American or Black, American Indian or Alaska Native, Asian, Native American or Pacific Islander, White, multiracial, and other race (defined as Portuguese, Dominican, Caribbean, or unspecified). For the purpose of randomization only, race and ethnicity were then used to categorize participants as either non-Hispanic White or from an underrepresented racial or ethnic group (i.e., anyone other than non-Hispanic White individuals).

### Interventions and Determination of Early WL Response

A detailed description of both the online WL program and telephone coaching intervention have been reported previously.^14^ The interventions are described briefly below.

### Online WL Program

All participants received a 12-month, online behavioral WL and WL maintenance program that has been rigorously tested^5,11,13,15^ and modeled after the Action for Health in Diabetes (Look AHEAD) trial behavioral intervention approach.^16^ Individuals were given a daily calorie intake goal (1200-1800 kcals/day), a moderate-intensity physical activity goal (progressed to ≥150 minutes/week), and an overall WL goal of 10% or greater. During the WL phase (months 1-4) participants were asked to watch weekly multimedia lessons and monitor daily calorie intake, exercise minutes, and weight on the study website. They were provided with weekly, computer-generated personalized feedback on their reported data. During the WL maintenance phase (months 5-12), the prescribed goal was to stay at or below their 4-month weight; however, participants could continue to lose weight if desired. In this phase, they received monthly multimedia lessons and were asked to self-monitor 1 week each month (more frequently if desired); computer-generated feedback was provided monthly.

### Criteria for Classification of Early Suboptimal Response

Percent WL at the end of month 1 was used to classify individuals as having suboptimal response (<4% WL) or initial response (≥4% WL). This was calculated using self-reported weights from the study website (shown to be associated with objective weights^17^). If a 1-month weight was not entered, research staff contacted the participant to obtain a weight. Those missing a 1-month weight (15 participants) were excluded from all analyses. Justification for using a 4% threshold has been described elsewhere.^14^ In short, using a higher threshold than what is typically used to define early nonresponse (ie, 2%), allowed for moderator analyses to examine for whom each dose of coaching was most effective (eg, those with the lowest 1-month WL [<2%] or those with modest 1-month WL [2%-4%]), a secondary aim, which is important from a treatment-matching perspective. Furthermore, 1 month was chosen as the time point for assessing early response because (1) prior studies have shown associations of 1-month WL with longer-term WL^9,11,13,18^ and (2) waiting too long to intervene may impede rescue efforts because individuals may already be disengaged.^19,20,21^

### Telephone Coaching Intervention

Participants classified as having suboptimal response and randomized to brief coaching received weekly coaching calls for 3 weeks (delivered during weeks 5-7), and those randomized to extended coaching received weekly coaching calls for 12 weeks (delivered during weeks 5-16). Individuals with suboptimal response who were randomized to the control group did not receive any telephone coaching. Initial responders, independent of randomization assignment, did not receive any coaching. Initial coaching calls were approximately 45 minutes and follow-up calls were approximately 15 minutes; these durations were chosen to maximize the efficiency of calls (ie, ample time for establishing rapport and addressing all intervention components while minimizing cost).

The coaching protocol was guided by the Supportive Accountability framework,^22^ which posits that human support increases program engagement when a coach is seen as trustworthy, benevolent, and having expertise. Therefore, a primary focus of the first coaching call was to establish rapport with the participant by asking about weight history, reasons for joining the program, and treatment goals. Additionally, on the first call, the coach and participant worked together to develop an individualized meal plan. On each call, self-monitoring data were discussed, continued intervention engagement was encouraged, barriers were identified, problem solving strategies were employed, education was provided, and small specific goals were set.

### Intervention Fidelity

Coaching calls were audio-recorded to assess treatment fidelity and 10% were randomly selected on a quarterly basis and reviewed by 1 of the authors (C.A.P). A fidelity checklist assessed whether coaches discussed weight, self-monitoring, calorie intake, meal plans, physical activity, video lessons, and set a specific, measurable, achievable, relevant, and time-bound goal. Coaching call length was also checked for consistency with study guidelines. Fidelity remained above the predetermined threshold (≥80%) throughout the study, no one common checklist component was missed, and average coaching call lengths were as intended.

### Cost Analyses

Costs were estimated from an organizational perspective, reflecting the costs that a health care system would pay to provide supplemental coaching to individuals already enrolled within online WL treatment. See the eMethods and eTable in Supplement 2 for a full description of the cost analyses. The cost per participant was calculated by summing all coaching costs within each treatment group and dividing by the number of participants randomized to that treatment group (regardless of early WL response). The incremental cost-effectiveness ratio (ie, cost/kilogram of WL) was calculated as the difference in cost per participant between a coaching intervention group and control group, divided by the difference in kilograms of WL observed.

### Assessment of Body Weight

To examine both the immediate and longer-term effects of coaching on WL, body weight was objectively assessed at baseline, 4 months (immediately following completion of all coaching), and 12 months. Research staff obtained in-person weights from all participants, with the exception of 38 participants (12 participants in the control group, 14 participants in the brief coaching group, and 12 participants in the extended group) who provided self-reported weights at 12 months due to state-wide shutdowns associated with the COVID-19 pandemic. Percent weight change from baseline to 4- and 12-month follow-up was calculated and the percentage of participants achieving 5% WL or greater was determined.

### Assessment of Intervention Engagement

Engagement with the WL program was assessed via the percentage of total video lessons viewed and days that weight and calorie intake were reported over 4 months (out of 16 possible lessons and 112 days) and 12 months (out of 24 possible lessons and 168 days). The coaching call completion rate was calculated as the total calls completed divided by the number of calls prescribed.

### Statistical Analysis

The study sample (including baseline demographics and anthropometrics) and intervention engagement were described using descriptive statistics. Statistical confounders were identified using correlation analysis from a candidate set identified based on the literature (including biological sex and enrollment relative to the COVID-19 pandemic).

Using a longitudinal mixed-effects model with participant-specific intercept, we examined intervention effects on percent WL at 4 and 12 months adjusting for confounders (only biological sex was retained in the adjusted model as a covariate). All analyses were run on the quasi–intent-to-treat sample with randomized participants who provided 1-month weight included in the analysis. Models used a likelihood or quasi-likelihood approach to estimation and, thus, were able to produce consistent estimates of the treatment effects using all available data without explicitly imputing missing outcomes. We compared effects with a multiple-imputation approach, and with no differences in the pattern of findings, we present the likelihood-based approach below.

Weight-change analyses were done in 2 ways; the primary analyses were done on the full sample of participants who had 1-month weight data; secondary analyses were done on the subgroup of participants who had suboptimal WL at 1 month. Because data were longitudinal and clustered within participants, standard errors were adjusted to account for this design feature. Models adjusted standard errors for repeated measures of the outcome within participant. Intervention was effect-coded to allow for all pairwise comparisons between treatment groups (eg, extended vs brief coaching, extended coaching vs control, and brief coaching vs control) using a single longitudinal model. Sensitivity analyses were used to examine whether the pattern of findings differed when self-reported weight (38 participants) was recoded as missing. All point estimates were in the same direction and did not significantly differ in magnitude; thus, presented data include both self-reported and objectively measured weights. Models did not assume equal retention between groups.

Intervention effects on the binary outcome (achievement of ≥5% WL at 4 and 12 months) was examined using a longitudinal regression model implemented with generalized estimating equations with robust standard errors. A logit link function was specified, resulting in effect sizes captured by odds ratios (ORs) and corresponding 95% CIs. Models included effects of intervention group (effect-coded), time, and intervention × time.

Longitudinal mixed-effects models as described above were used to examine whether 1-month WL was a moderator of the treatment effect. The model included the main effects of intervention, the potential moderator, time, and all 2- and 3-way interactions. All models were run in SAS software version 9.3 (SAS institute) and significance level set at a 2-sided *P* < .05 a priori.

## Results

**Figure 1.**
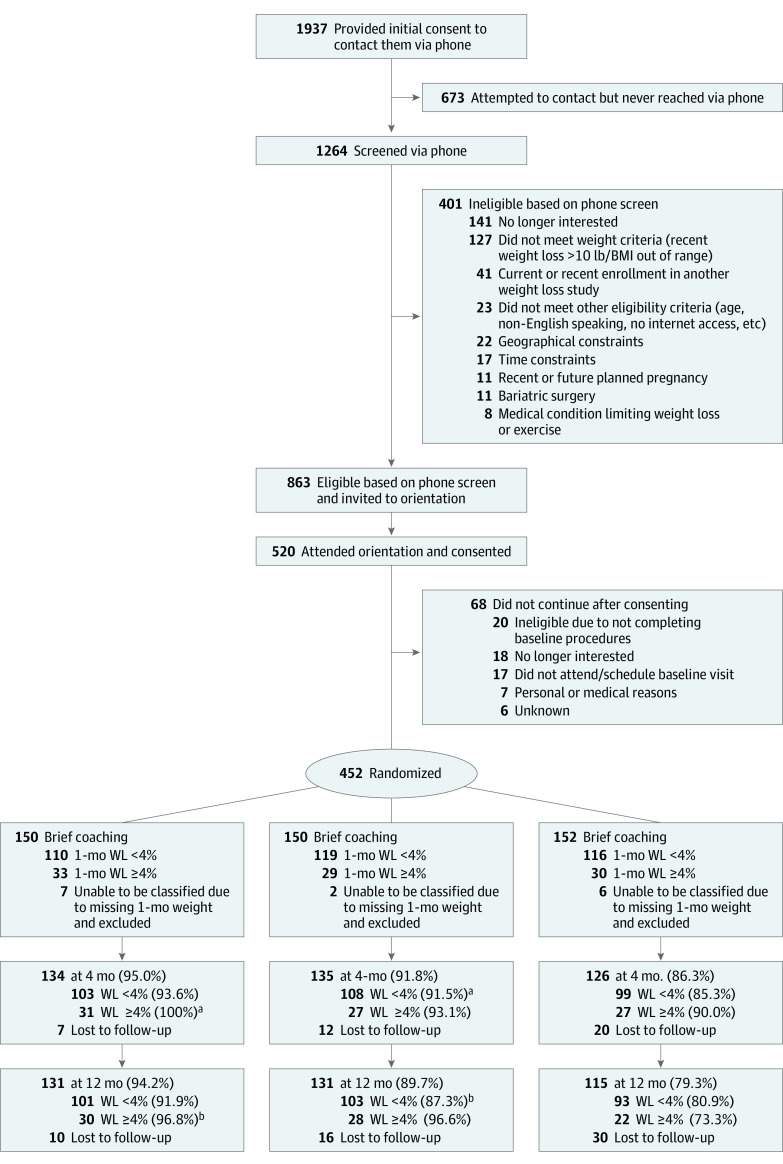
Flow Diagram BMI indicates body mass index; WL, weight loss. ^a^A total of 2 participants in the brief group and 1 participant in the extended group were unable to continue due to pregnancy or medical condition and were not counted toward percentages. ^b^A total of 2 participants in the brief group, 1 participant in the extended group, and 1 participant in the control group were unable to continue due to pregnancy or medical condition and were not counted toward percentages.

The analyzed sample included 437 participants (mean [SD] age, 50.8 [11.4] years; mean [SD] BMI, 34.6 [5.0]; 305 female [69.8%] and 132 male [30.2%]), with 148 randomized to extended coaching, 143 assigned to brief coaching, and 146 assigned to control (Figure 1 and Table). Of all participants, 292 [66.8%] had a bachelor’s degree or higher. Across groups, retention at 4 months (395 participants [91.0%]) and 12 months (377 participants [87.1%]) was high. There were significant between-group differences in retention at 4 months (brief coaching, 134 participants [95.0%]; extended coaching, 135 participants [91.8%]; control, 126 participants [86.3%]; *P* = .03) and 12 months (brief coaching, 131 participants [94.2%]; extended coaching, 131 participants [89.7%]; control, 115 participants [79.3%]; *P* < .006).

**Table.** Descriptives of Study Sample by Group

Variable	Participants, No. (%)			
	Total (N = 437)	Control (n = 146)	Brief (n = 143)	Extended (n = 148)
Biological sex				
Female	305 (69.8)	103 (70.5)	101 (70.6)	101 (68.2)
Male	132 (30.2)	43 (29.5)	42 (29.4)	47 (31.8)
Age, mean (SD), y	50.8 (11.4)	48.5 (11.4)	52.2 (11.5)	50.7 (10.9)
Body mass index, mean (SD)^a^	34.6 (5.0)	34.7 (5.3)	34.4 (4.8)	34.7 (5.1)
Weight, mean (SD), kg	96.6 (17.9)	96.8 (18.9)	96.5 (17.1)	96.4 (17.8)
Ethnicity				
Hispanic	56 (12.8)	23 (15.8)	15 (10.5)	18 (12.2)
Non-Hispanic	381 (87.2)	123 (84.2)	128 (89.5)	130 (87.8)
Race				
African American or Black	39 (8.9)	14 (9.6)	15 (10.5)	10 (6.8)
American Indian or Alaska Native	0	0	0	0
Asian	7 (1.6)	2 (1.4)	2 (1.4)	3 (2.0)
Native American or Pacific Islander	0	0	0	0
White	353 (80.8)	117 (80.1)	116 (81.1)	120 (81.1)
Multiracial	17 (3.9)	4 (2.7)	5 (3.5)	8 (5.4)
Other^b^	21 (4.8)	9 (6.2)	5 (3.5)	7 (4.7)
Education level				
High school or lower	27 (6.1)	6 (4.1)	10 (7.0)	11 (7.4)
Vocational training	16 (3.7)	6 (4.1)	4 (2.8)	6 (4.1)
Some college (<4 y)	102 (23.3)	34 (23.3)	31 (21.7)	37 (25.0)
College degree	166 (38.0)	63 (43.2)	53 (37.1)	50 (33.8)
Graduate or professional degree	126 (28.8)	37 (25.3)	45 (31.5)	44 (29.7)
1-mo weight loss				
<2%	164 (37.5)	59 (40.4)	50 (35.0)	55 (37.2)
2%-4%	182 (41.6)	58 (39.7)	59 (41.3)	65 (43.9)
>4%	91 (20.8)	29 (19.9)	34 (23.8)	28 (28.9)

^a^ Body mass index was calculated as weight in kilograms divided by height in meters squared.

^a^ Other race includes Portuguese, Dominican, Caribbean, or unspecified (ie, not falling into any other racial categories but participant did not elaborate further).

### Intervention Engagement

**Figure 2.**
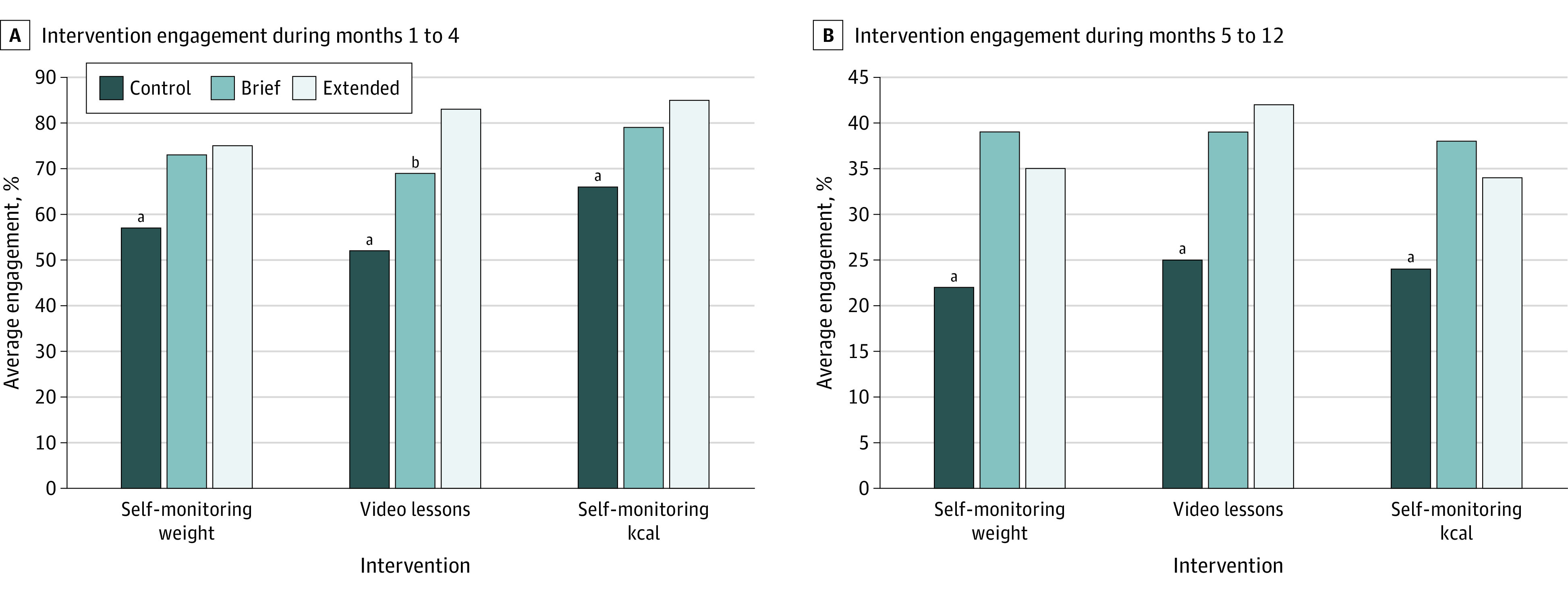
Intervention Engagement The figure shows intervention engagement during months 1 to 4 (A) and months 5 to 12 (B). ^a^Statistically significant difference between control and both brief and extended interventions (*P* < .05). ^b^Statistically significant difference between brief and extended intervention (*P* <.05).

Coaching call completion rate was 97% (321 of 330 calls) in the brief coaching group and 94% (1337 of 1428 calls) in the extended coaching group (*P* = .12) and coaching fidelity was 85%. Intervention engagement data are presented in Figure 2. Over months 1 to 4 and months 5 to 12, both the brief and extended coaching groups watched more video lessons and self-monitored weight and calories on a greater percentage of days compared with the control group. The extended coaching group also watched significantly more video lessons than the brief coaching group during months 1 to 4.

### WL Outcomes

**Figure 3.**
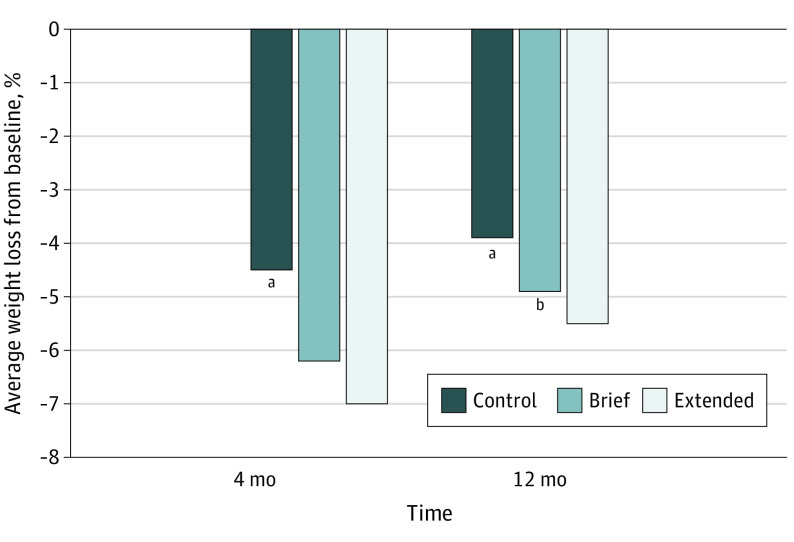
Percent Weight Change by Treatment Group at 4 and 12 Months ^a^Statistically significant difference between control and both brief and extended interventions (*P* < .05). ^b^Statistically significant difference between brief and extended intervention (*P* <.05).

**Figure 4.**
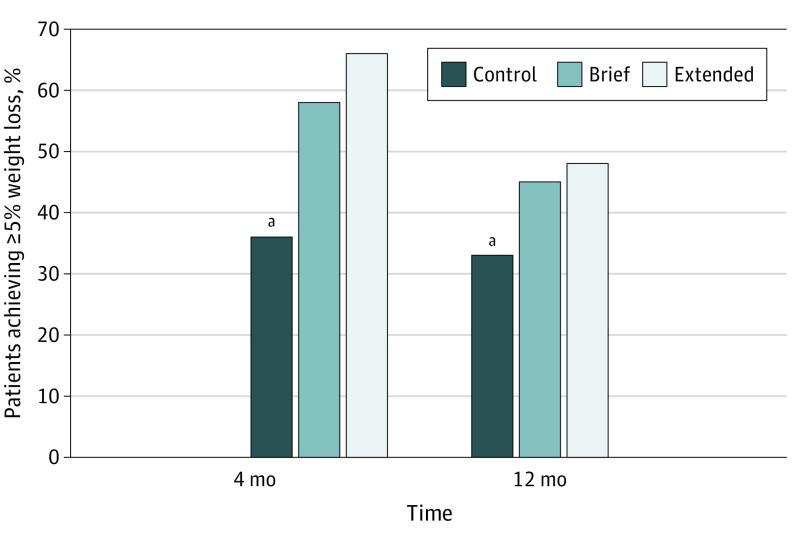
Percentage of Participants Achieving 5% Weight Loss or Greater by Treatment Group at 4 and 12 Months ^a^Statistically significant difference between control and both brief and extended interventions (*P* < .05).

At the end of the WL phase (ie, 4 months), percent WL was significantly greater in the extended coaching group (mean [SD] WL, −7.0% [5.1%]) and brief coaching groups (mean [SD] WL, 6.2% [4.7%]) vs control group (mean [SD] WL, (−4.5% [4.7%]) (*P* < .001) (Figure 3). At the end of the WL maintenance phase (12 months), WL was significantly greater in the extended coaching group (mean [SD] WL, (−5.5% [6.7%]) vs the control group (mean [SD] WL, (−3.9% [7.4]) (*P* = .03); but there was no significant difference in WL between the brief coaching group and control group. The extended and brief coaching groups did not differ in percent weight loss at 4 or 12 months. The proportion of participants achieving 5% or greater WL at 4 months was greater in the extended coaching group (89 participants [65.9%]) and brief coaching group (79 participants [58.5%]) vs control group (46 participants [36.5%]) (*P* < .001) (Figure 4). At 12 months, a similar pattern was observed for achievement of 5% WL or greater (extended coaching, 63 participants [48.1%]; brief coaching, 61 participants [45.9%]; control, 38 participants [32.8%]; *P* < .03) (Figure 4).

Secondary analyses examined percent WL among 346 individuals with suboptimal response only (79.2% of the analyzed sample). Similar to the entire analyzed sample, the same pattern of findings was observed at 4 and 12 months (eFigure 1 in Supplement 2). An additional secondary aim assessed whether early WL (ie, 1-month) was a significant moderator of the treatment effect. Findings revealed that among those with 1-month WL less than 2%, the extended coaching group outperformed the brief coaching group at 4 and 12 months, with no difference observed between the extended and brief coaching groups among those who lost 2% to 4% at 1 month (eFigure 2 in Supplement 2).

### Cost Analyses

The cost per participant of adding supplemental coaching to a pre-existing online WL program was $65.62 in the brief coaching group (equivalent to $13.18/kg) and $167.69 in the extended coaching group (equivalent to $30.76/kg); the cost of the control group was $0 because no coaching was provided. Incremental cost-effectiveness analyses indicated that over 12 months, the cost per additional kilogram of WL, beyond that of the control group was $50.09 in the brief coaching group and $92.65 in the extended coaching group.

## Discussion

To our knowledge, this is the first fully powered randomized clinical trial to assess whether WL is improved by providing additional support to individuals with suboptimal response within online WL treatment. Consistent with hypotheses, both brief and extended coaching resulted in better program engagement and more participants achieving 5% or greater WL at 4 and 12 months compared with no coaching. Percent WL findings were partially supported by hypotheses in that both brief and extended coaching lost more weight than control at 4 months, but only extended coaching was significantly different than control at 12 months. Also noteworthy is the fact that retention rates were lower in the control vs coaching groups at both 4 and 12 months, suggesting that additional intervention, human support, or a combination of both, for individuals with suboptimal response may help reduce dropout rates within automated online programs.

Overall findings suggest that adapting an intervention forindividuals with suboptimal response is an effective approach to enhance WL among those in an online program. More specifically, these results suggest that for maximizing long-term WL, extended coaching may be most effective. However, if there are cost concerns, brief coaching is more cost-effective. Furthermore, our results suggest that 1-month WL could also be used to guide decisions regarding the optimal supplemental intervention. Extended coaching was more effective in those with 1-month WL less than 2%, and brief coaching was sufficient in those with 1-month WL between 2% and 4%. Interestingly, this finding contrasts with a prior study^13^ which found that brief coaching doubled WL in those with 1-month WL less than 2.3%. Possible explanations for discordant findings may be the assessment length (12 weeks vs 4 to 12 months) or participants being less enthusiastic about 3 weeks of coaching when 12 weeks was offered.

This adaptive intervention approach offers a cost-effective alternative to obesity treatment. The cost per participant for adding coaching for individuals with suboptimal response within our online WL program (brief, $65.62; extended, $167.69) is far less than group-based intensive lifestyle interventions (costs range from $706-$2865 per participant^23,24,25^). Similarly, a 2018 systematic review^26^ reported costs of $134/kg with Weight Watchers, $444/kg with Jenny Craig, $251/kg with Orlistat, and $2102/kg with Saxenda—all greater than the cost of adding coaching to a preexisting online WL program (brief, $13.18/kg; extended, $30.76/kg). Considering recent growth in the health coaching market, establishment of *Current Procedural Terminology* codes for health and well-being coaching, uptake of coaching within health care systems, and expansion of reimbursement coverage for telehealth services,^27,28^ this adaptive intervention model has potential for sustainable implementation into health care networks or primary care settings. For example, primary care practices could partner with health coaching organizations, or large physician offices could implement this type of program into patient-centered medical home initiatives. However, pragmatic trials (ie, trials conducted in a clinical practice setting) are first needed to determine whether similar findings are observed in these types of settings.

### Limitations

While this trial has notable strengths (eg, large sample size, comparison of coaching doses, and inclusion of cost-effectiveness analyses), potential limitations include self-reported weights at 1 month (although objective weights would increase cost and reduce translatability), and a study sample not representative of the population (74.4% were non-Hispanic White, 69.8% female, 66.8% with bachelor’s degree or higher), which limits generalizability. Furthermore, although common in the field, weight regain occurred between 4 and 12 months, which raises questions regarding the optimal dose and timing for offering supplemental coaching and whether additional coaching should also be provided later in the program for those not meeting prespecified WL thresholds. Finally, as mentioned above, the use of a 4% WL threshold to determine early suboptimal response allowed for the evaluation of an important secondary aim of this trial; however, the use of this high threshold meant that 79.2% of individuals were classified as having suboptimal response. Program goals, cost constraints, and dose of coaching should be considered when selecting an early WL threshold in future studies.

## Conclusions

The findings of this study suggest that providing coaching for individuals with suboptimal response within online obesity treatment improves WL and program engagement. This adaptive intervention model is a cost-effective and scalable treatment alternative to traditional obesity management. Future pragmatic trials are needed to test this treatment approach within a clinical setting (eg, a health care system) and continued research should examine how to optimize coaching efforts within an automated WL program. Particular emphasis should also be placed on recruitment of more diverse and representative samples of adults with obesity.

## Notes

Supplement 1.
Trial Protocol

Supplement 2. **eMethods.** Description of Cost Analyses **eTable.** Summary of Coaching Costs **eFigure 1.** Examination of Percent Weight Change at 4 and 12 Months Among Early Suboptimal Responders Only (n=346) **eFigure 2.** Examination of 1-Month Weight Loss as a Treatment Moderator of Weight Loss at (a) 4 Months and (b) 12 Months by Randomization Assignment

Supplement 3.
Data Sharing Statement
