# Supplementary material for: An Adaptive Telephone Coaching Intervention for Patients in an Online Weight Loss Program: A Randomized Clinical Trial
Source: JAMA Netw Open. 2024 Jun 7;7(6):e2414587. doi: 10.1001/jamanetworkopen.2024.14587 (PMC11161849; doi:10.1001/jamanetworkopen.2024.14587)
Supplement: Supplement 1. — Trial Protocol [file jamanetwopen-e2414587-s001.pdf]

DIAL Now Trial Protocol
(Disseminating Internet-based Approaches to Lifelong change)
4/4/20

Background.
Internet-based weight loss (WL) programs are effective for many, but a significant number of individuals fail to achieve a clinically significant WL and may require a more personalized treatment approach. One strategy for improving Internet-delivered WL (IDWL) treatment, while still limiting delivery costs, is to use a stepped-care intervention, in which all individuals receive a standardized Internet program and a more intensive intervention is reserved for those with suboptimal WL outcomes. Our research team has demonstrated that the provision of a brief period of individualized coaching to early non-responders enrolled in IDWL treatment significantly improved intervention adherence and post-treatment WL, when compared to early non-responders who received no coaching. The current study builds on these findings and examines whether WL can be further improved through the use of extended coaching, whether these effects are sustained once the phone coaching (PC) is removed, whether these interventions are effective from a cost perspective, and the mechanisms through which PC affects intervention engagement and WL.

Study Design and Overview. 450 participants will be randomized at baseline to 1 of 3 groups: 1) IDWL only (‘Control’; n=150), 2) IDWL+Brief PC for early non-responders (‘Brief’; n=150), or 3) IDWL+Extended PC for early non-responders (‘Extended’; n=150). Randomization will occur in equal numbers based on permuted block randomization procedure with small, random sized blocks. Randomization will be stratified by sex and race/ethnicity (non-Hispanic White vs. Other).

An overview of the study design is shown in Figure 4. Participants will be recruited in multiple cohorts and the study will be conducted over a 5-year period. All individuals will receive a 4-month IDWL program, followed by an 8-month IDWL maintenance program. Participants will report their weight weekly on the study website and 4-week WL will be used to classify individuals as initial responders (4-wk WL ≥4%) or early non-responders (4-wk WL <4%). Initial responders will continue the IDWL program but will not receive any additional intervention, while treatment for early non-responders will be dependent upon randomization assignment. Early non-responders randomized to ‘Control’ will not receive PC while early non-responders randomized to ‘Brief’ will receive weekly PC for 3 weeks (weeks 5-8), and early non-responders randomized to ‘Extended’ will receive weekly PC for the remainder of the WL program (weeks 5-16). During the WL maintenance phase (months 5-12), monthly video lessons will be provided and participants will continue to receive automated feedback monthly; however no additional PC will occur during this time. Assessments will be performed at baseline, post WL treatment (4 months) and following the 8-month, no-contact WL maintenance period (1 year). Body weight, intervention adherence, diet, physical activity, and psychosocial factors will be assessed.

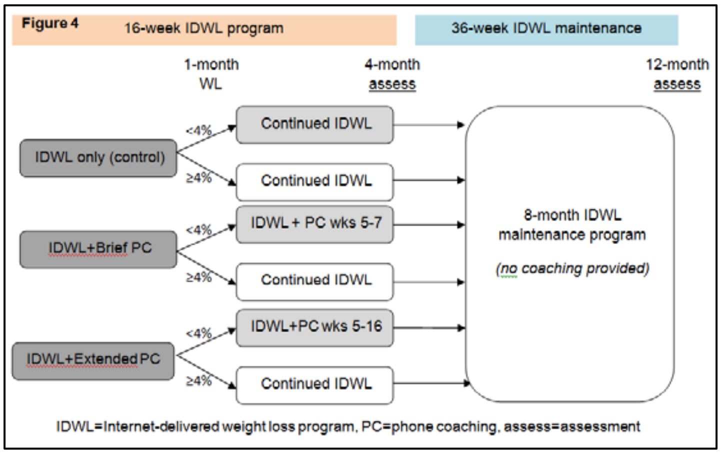

Study Aims.

Primary aim: To compare treatment effects on weight outcomes at 4 and 12 months. The primary weight outcome is percent weight change from baseline to 4 and 12 month follow-ups, and the secondary weight outcome is the percentage of participants achieving a clinically significant WL (≥5% WL). *It is hypothesized that WL will be: Extended > Brief > Control.* We will also examine treatment effects on primary and secondary weight outcomes among early non-responders only.

Secondary aims: (1) To examine the incremental cost per kg of WL in Brief, Extended, and Control. (2) To compare Brief, Extended and Control on intervention engagement (e.g., diet quality, physical activity, self-

monitoring, video lessons viewed) and to examine whether intervention engagement mediates the effects of the intervention on WL. (3) To examine whether 4-week WL moderates the effect of the interventions on longer-term WL. By using a 4% WL threshold to classify early non-responders, we can both continuously and categorically (e.g., <2% vs. 2-4%) examine whether there is an interaction between early WL and the efficacy of the interventions.

Exploratory aim: To explore mechanisms through which PC may affect engagement and WL, including perceived support, self-efficacy and intrinsic/extrinsic motivation and consider other moderators (e.g., gender, race/ethnicity).

Recruitment. Participants will be recruited through advertisements in local newspapers, direct mailings, Internet and television ads, and by using LifeChart through a generated Report. Individuals will be instructed to: 1) call the WCDRC to see if they are eligible to participate in this study and a member of the research staff will conduct a formal phone screen with them via the phone (see attached phone screen). 2) go to our study website to learn more about the study and to complete an initial online screener via RedCap (see attached for text describing the study and online screener). Those who appear to be eligible based upon the online screener will be called by a member of our research staff in which the remaining questions from the phone screen will be asked by a member of our research staff. All eligible participants will be scheduled for an orientation session either in-person, via phone or video call where they will learn more about the study and if interested, informed consent will be obtained in-person or electronically using Redcap.

Participants. 450 weight loss seeking men and women will be recruited for this study. Participants will be between the ages of 18 and 70, have a BMI between 25 and 45 kg/m^2, and have regular (i.e., daily) access to the Internet, defined as owning a smartphone with Internet capabilities, and/or having a broadband or wi-fi connection at home or work. Additional exclusionary criteria can be found in the *Protection of Human Subjects* section and Clinical Trials Section 2.2. *Eligibility Criteria.*

Internet-based weight loss program. All participants, independent of randomization assignment or early non-responder status, will receive a 4-month, Internet-delivered behavioral WL program followed by an 8-month, minimally intensive, IDWL maintenance program. Prior to the start of the program, participants will be taught how to navigate the study website and will be given the opportunity to ask questions. The goal of the program is to induce a 1-2 lb WL per week. To assist in achieving this goal, individuals will be given a daily calorie intake goal of 1200-1800 kcal/day depending upon starting weight and a physical activity (PA) goal which progresses to 250 min/week of moderate-intensity PA. Using the self-monitoring platform on the website, participants will be instructed to record their body weight, calorie intake, and PA daily over the 16-week period. If participants do not own a scale, one will be provided. Computer-generated, personalized feedback will be provided weekly based upon these self-report data. Further, participants will be instructed to view a 10-15 min multimedia lesson weekly. These lessons are modeled after the Look AHEAD Trial[80] and example lesson topics including strategies for increasing PA, eating out at restaurants, and problem solving. Moreover, the study website gives participants access to weekly recipes and additional tip sheets offering useful strategies for healthy eating and regular PA (e.g., strategies for meal planning, dealing with negative emotions, or exercising safely).

Following the completion of the 16-week WL program, all participants will transition to a no-contact, minimally intensive WL maintenance program. During months 5-12, participants will have the opportunity to view monthly video lessons which focus on strategies for successful WL maintenance (e.g., relapse prevention, goal setting, overcoming barriers, etc.) and they will be instructed to continue to self-monitor their weight, dietary intake, and physical activity minutes on the study website for one week every month. Automated feedback will only be provided monthly and will be tailored based upon whether the participant lost, gained, or maintained WL. No phone coaching will occur beyond 4 months.

Identification of early non-responders. Percent WL at the end of week 4 will be used to classify individuals as early non-responders (<4%) or initial responders (≥4%). This will be calculated using self-reported weight at baseline and weight at the end of the week 4. If a participant did not report a weight at week 4, an automated email will be sent prompting them to log their weight immediately. If after 3 email attempts a weight is still not

recorded, the participant will receive a telephone call. Participants still missing 4-week weight data will be unable to be classified as an initial responder or early non-responder and thus will only be included in the analyses involving the aggregate sample. To reduce potential bias and enhance scientific rigor, participants will not be informed of their randomization assignment or the criteria used to determine who will receive PC. Instead, all participants will be told that they will receive an IDWL program and have the possibility to receive a brief period of PC or PC for the entire duration of the WL portion of the program, and that they will be contacted if they were selected to receive the PC.

Phone coaching for early non-responders. Early non-responders randomized to 'Brief' will receive weekly PC for 3 consecutive weeks (delivered during weeks 5-7), while early non-responders randomized to 'Extended' will receive weekly PC for the remainder of the WL program (delivered during weeks 5-16). Coaches will be dieticians, exercise physiologists, or interventionists at the WCDRC with previous intervention experience in behavioral WL programs. All coaches will be trained and supervised by Dr. Pellegrini (Co-I). Training will be consistent with that proposed in the Supportive Accountability model and will include basic motivational interviewing and problem solving strategies, establishing trust and rapport with the participant, practical solutions for barriers common to early non-responders, and individualized meal planning methods. The initial PC session will be approximately 45 minutes in duration and all remaining sessions will be approximately 10-15 minutes. The content material of the coaching calls will be similar to that provided in our pilot study[11] and is guided by the Supportive Accountability Model which posits that human support (via coaching) improves adherence to web-based treatment programs through accountability to a coach who is viewed as being trustworthy, benevolent, and having expertise developed by Mohr and colleagues[39]. The TeleCoach Manual[38], which was developed within the context of the Supportive Accountability framework, will be adapted for WL and used in the proposed study.

The overarching goal of the coach is to help the participant 'jump start' his or her WL through the provision of a supportive environment. Thus, a large portion of the initial PC session will be spent establishing rapport and focusing on each of the following elements: understanding the context of the participant's life, eliciting their coping and treatment history and hopes for treatment, providing feedback and brief psychoeducation, identifying potential barriers and problem solving, reviewing the program website, and eliciting commitment to the process of the coaching calls (see Clinical Trials 4.2.a for more details). The goal of the follow-up phone calls is to use motivational interviewing principles to keep the participant motivated, elicit commitment, and problem solve any barriers to intervention adherence or achievement of program goals. Prior to the phone call, coaches will review the participant's website logins and self-monitoring data. These follow-up sessions will begin with a brief check-in and conclude by setting a SMART (Specific, Measurable, Achievable, Results-focused, Time-bound) goal for the upcoming week. During each PC session, participants will also be encouraged to continue to self-monitor on the study website and view weekly video lessons. Further, during the last week of PC for 'Extended' participants, a portion of the phone call will focus on strategies for successful WL maintenance and maintaining accountability and motivation following the cessation of PC.

While the structure of the coaching calls will be similar across participants, the content will be tailored to the individual. Coaches will use goal setting, problem solving and motivational interviewing strategies to help participants adhere to the intervention and achieve program goals; thus having the ability to address both typical barriers as well as non-traditional barriers to WL. Our preliminary data highlight several common barriers among early non-responders. Some examples are frequent eating out, feelings of being overwhelmed (too busy), underestimation of caloric intake, and greater than normal effort required to stay on track. Thus, coaches will be provided with training and resources highlighting potential strategies for overcoming each of these barriers and they will be trained in how to convey this information with the participant (see *C.1.d* above).

Coaches will also be trained in developing individualized meal plans. In our pilot study, coaches worked with the participants to develop an individualized meal plan for the upcoming week that relied heavily on pre-packaged foods (e.g., meal replacement products, frozen entrees, single size portion snacks, etc) and fruit and vegetable consumption. Meal planning with a coach can reduce the amount of effort required when counting calories and self-monitoring, ensure that the participant achieves his/her calorie intake goal, save time for those who are busy, and reduce restaurant eating and/or lead to better food choices while eating out, thereby addressing many of the common barriers mentioned above. These meal plans will be tailored to each participant's food preferences in order to promote adoption of this meal plan and revised weekly if needed.

To ensure rigor and reproducibility of our PC sessions, coaches will be provided with standardized manual to ensure that critical intervention elements are discussed during each PC session, coaches will be trained by Dr. Pellegrini, and calls will be audio recorded and treatment fidelity will be assessed (see C.11.b).

Assessment components. Assessments of weight, intervention engagement (diet, physical activity, adherence), and psychosocial factors (perceived support, self-efficacy, motivation) will occur at baseline, 4, and 12 months. Brief descriptions of these measures are provided below.

Weight, height, and body mass index. Objective measures of weight and height will be performed by a staff member. Calculations of body mass index, absolute weight change in kg, and percent weight change from baseline will be calculated. Further the percentage of participants in each treatment group achieving a clinically significant WL (≥5%) will be determined. Percent WL at the end of week 4 will be calculated using self-reported weight obtained from the study website at baseline and 4 weeks, and these data will be used to categorize participants as early non-responders or initial responders.

Intervention engagement. Diet, physical activity (PA), and intervention adherence are important weight control behaviors which can influence body weight.

- **Physical activity** will be objectively-measured for 1 week at baseline, 4, and 12 months using the newest generation of Actigraph accelerometers (Actigraph GT9X Link). Participants will receive the Actigraph at their scheduled assessment visit and will be instructed to wear the accelerometer on their waist during all waking hours, exclusive of bathing and swimming, for 7 consecutive days at each assessment period. This monitor uses a validated triaxial accelerometer and proprietary data filtering technology to estimate free-living PA and sedentary behaviors[81-83]. Accelerometer data will be processed and analyzed using Actigraph's ActilLife software. Data will be considered valid if the monitor was worn for ≥10 hours on ≥4 days (including ≥1 weekend day). Intensity will be assessed as average counts per minute (cpm) and previously validated cut points will be used to identify bout-related moderate-to-vigorous intensity PA (≥1952 cpm accumulated in bouts ≥10 min) and sedentary time (<100 cpm). Minutes per day spent in moderate-to-vigorous intensity PA and sedentary behaviors will be calculated.
- **Diet** will be assessed using the web-based Dietary Screener Questionnaire (DSQ) developed by NCI[84]. This questionnaire was originally validated using recall data form the National Health and Nutrition Examination Survey. The DSQ is a 26-item screener that asks about the frequency of consumption in the past month of selected foods and drinks, such as fruits and vegetables, dairy/calcium, added sugars, whole grains/fiber, red meat, and processed meat. The Dietary Screener will allow us to examine diet quality and changes in dietary factors targeted by the intervention and encompassed within healthy eating lesson material (e.g., reducing intake of sugar sweetened beverages, baked good and other refined carbohydrates and increasing intake of fruits, vegetables and fiber. To assess for total energy intake, participants will report caloric intake via self-monitoring records.
- **Intervention adherence** will be assessed separately during the 4-month WL program and 8-month WL maintenance period. Outcome measures include the number of website logins, video lessons viewed, and the frequency of self-monitoring, all of which are generated automatically by the study website. The number of coaching calls completed will also be assessed for 'Brief' and 'Extended' early non-responders.

Psychosocial process measures. An exploratory aim of this trial is to examine psychosocial process measures which could mediate or moderate the effect of PC on adherence and WL.

- **Perceived support** will be measured in two ways. First, all participants will complete the *Important Others Questionnaire*[90] which measures the participants perception of the supportiveness of 'important others' which can be a family member, spouse, friend, or coach with respect to their diet and PA behaviors. This will allow for comparisons in perceived support between all three treatment groups which is important for meditational analyses. However, it is equally important to measure the strength of the coach-participant bond, as this is an important aspect of the Supportive Accountability model; thus those randomized to Brief or Extended will also complete the *Helping Alliance Questionnaire*[91]. This will allow for the exploration of whether the coach-patient bond differs between Brief and Extended, and whether the magnitude of alliance is associated with WL.
- **Self efficacy** for diet will be assessed using the *Weight Efficacy Lifestyle (WEL) Questionnaire*[92] which assesses one's confidence in their ability to avoid overeating when faced with certain situations. Self-efficacy

for PA will be assessed by the Self-*Efficacy for Exercise Scale*[93] which evaluates an individual's confidence to exercise when faced with a variety of barriers such as fatigue or a lack of time.

- **Motivation** will be assessed using the *Treatment Self-Regulation Questionnaire for Weight Management*[94] which assesses the degree to which an individual's motivation for weight management behaviors is relatively autonomous or self-determined.

Other weight-related factors: Self-compassion will be assessed using the *Self-Compassion Scale* which assesses the degree to which an individuals acts towards themselves when experiencing a difficult time. Perceived stress will be measured via the *Perceived Stress Scale*, which assesses the degree to which situations in one's life are appraised as stressful. Restrained and disinhibited eating will be assessed using the *Three-Factor Eating Questionnaire* which measures three dimensions of eating behavior. Reasons why behaviors are engaged in to manage weight will be evaluated by the *Reasons for Weight Management Questionnaire*. Weight bias and weight stigma will be assessed using the *Weight Bias Internalization Scale* and *Weight Stigma Questionnaire* respectively. Important weight-control behaviors will be assessed using the *Weight Control Strategies Questionnaire* which consists of 4 subscales: dietary choices, self-monitoring strategies, physical activity, and psychological coping. Weight loss goals will be evaluated using the *Goals and Relative Weights Questionnaire* and realistic weight loss will be evaluated using the *Realistic Weight Loss Questionnaire*. The *Paffenbarger Activity Questionnaire* (PAQ) will be used to assess leisure time physical activity and provides an estimate of minutes and calories expended per week in overall leisure time activity and in activities of light (5 kcal/min), medium (7.5 kcal/min), and high (10 kcal/min) intensity. All active participants will also be asked to complete a one-time questionnaire related to how their lives and weight loss efforts have been affected by the coronavirus. This questionnaire will be completed online by the participant using Redcap.

Participants will receive $50 for completion of the 4 month assessment and $75 for completion of the 12 month assessment. Cash will be distributed at an in-person visit, however, if those visits cannot occur participants can choose to either have a check mailed directly to them or have an amazon gift card emailed to them.

Cost and cost-effectiveness analysis. This study seeks to inform future WL dissemination efforts into healthcare and community settings; thus in addition to examining differences in WL between the 3 early non-responder treatment groups, it is also critical to evaluate the effectiveness of PC from a cost-perspective. All participants enrolled in this study will receive an identical IDWL and WL maintenance program. Once developed the cost of this automated program is minimal and the cost of implementing this program remains fixed, regardless of the number of individuals enrolled. Therefore, the current analyses focus on the absolute and relative costs of 'Brief' and 'Extended' PC for early non-responders, compared to 'Control' (no coaching).

The cost-effectiveness of adding PC for early non-responders enrolled in IDWL treatment will be assessed from both an organizational perspective (as this will drive adoption and impact potential scalability) and a societal perspective[95].The incremental cost-effectiveness ratio, defined as the difference in cost between the interventions, divided by the difference in their effect on WL, will be the primary cost-effectiveness outcome for this trial. This represents the average incremental cost associated with each additional kilogram lost in 'Brief', 'Extended', and 'Control'. We will also assess whether this cost differs by the degree of initial WL (e.g., 4-wk WL <2% vs. 2-4%), as it is possible that the cost-benefit of the different doses of coaching are dependent upon the magnitude of weight lost within the first 4 weeks of treatment. Costs will reflect the full costs of implementation (including training costs) and delivery of the proposed PC intervention. This includes overhead costs, such as office space for coaches and access to telephone lines, and interventionist costs which take into account the cost of interventionist training, intervention preparation, scheduling, and the completion of phone calls. Interventionist time will be valued using payroll records. Participant costs (used only in the analyses which assess cost from a 'societal' perspective) equal the value of time spent on the phone calls. Time will be measured by the interventionist and valued at the median wage. In addition to calculating the cost/kg of WL, we will also assess the absolute cost of the PC (i.e., total cost to implement and deliver the intervention) and the cost of the coaching per participant for 'Brief' and 'Extended' treatment groups.

Treatment fidelity. To ensure that the intervention is delivered as designed, phone coaches will adhere to treatment manuals and Drs Unick (PI) and Pellegrini (Co-I) will train the coaching staff. All PC sessions will be audio-recorded and 10% will be randomly selected and reviewed by a Dr. Pellegrini and her staff at the University of South Carolina using a session-specific fidelity checklist. Fidelity checks will occur on a quarterly basis. Fidelity checks will ensure good coaching practices and intended session content (e.g., meal planning, SMART goals). Further, the length of all PC sessions will be recorded to ensure that the amount of contact time is within the guidelines. If at any time fidelity is <85%, coaches will be retrained via videoconference by Dr. Pellegrini.

Data collection and management. All assessment data will be entered into the data management program by a member of the research staff. Range checks will be built into the data entry procedure to alert staff to data that requires additional clarification. Dr. Unick will conduct error checking and preliminary analyses of all data to ensure accuracy. Questionnaire data will be collected using REDCap (Research Electronic Data Capture), which is a secure, web-based application designed to support data capture for research studies and is hosted by Lifespan. It provides an intuitive interface for validated data entry, audit trails for tracking data manipulation and export procedures, and automated export procedures for seamless data downloads to common statistical packages. Only IRB approved research staff will have access to the REDCap platform and staff members will be granted access through a secure login provided by the study PI. Finally, Mr. Daniello (WL Application Programmer) will be responsible for preparing and maintaining the electronic data collection and storage system of all intervention data.

Statistical Analysis, Sample Size, and Power Estimation. With a sample size of n=450, the proposed study is fully powered to detect between group differences in both primary and secondary weight outcomes (e.g. % WL and % achieving clinically significant WL). Further, we have >70% power for all secondary aims. Full details regarding the power analysis, statistical approach, and methods for handling missing data can be found in the *Statistical Design and Power Section (*Clinical Trials 4.4*).* Dr. Dunsiger, the study biostatistician will provide guidance and oversee all aspects of data analysis plan.

STATISTICAL DESIGN AND POWER

Sample Size Considerations

The proposed study is fully powered for the Primary Aim which includes both primary and secondary weight outcomes (e.g. % WL and % achieving clinically significant WL). Consistent with expert opinion[103,104], we integrated several sources of evidence to determine an expected effect size for the proposed study. Our goal was to be powered for both treatment effects among the aggregate sample of participants (all randomized participants), as well as among early non-responders only. Based upon unpublished data from our IDWL programs, we anticipate 75% of participants will be early non-responders. These data suggest a mean WL of 3.7% (SD=3.8%) among early non-responders, and our pilot data[11] suggest brief coaching among early non-responders will correspond to an additional 2% WL (or mean of 5.7% WL). Further greater contact time is associated with greater WL[40-45], thus we anticipate the Extended PC will result in an additional 2% WL among early non-responders (mean 7.7%). Similarly, for the aggregate sample (all randomized participants) we expect weight losses of 4.7% (Control), 6.2% (Brief), and 7.7% (Extended) at 4 months. These estimates are based upon our previous data suggesting that 25% of participants will be initial responders, and that mean WL among this subgroup will be 7.7%. Given the above estimates and a two-sided alpha=0.05, we would need N=360 participants (120/group) to have 80% power to test the primary aim among both the aggregate sample and early non-responders only. However, given the known risks of powering on pilot data, as well as the longer follow-up than our pilot work, we have chosen to inflate the proposed sample size to N=450 (150/arm). With the increased sample size of N=450, we have 80% power to detect a 1.8% WL difference between treatment groups for early non-responders only, and 1.25% WL for the aggregate sample. Although our goal was to power for the primary study aim, a sample size of 450 also allows us to be reasonably powered for secondary aims (>70% for all secondary aims, assuming small-medium effect sizes, f^2=0.08). Our exploratory aims are considered hypothesis generating, rather than hypothesis testing, and thus we do not present a priori power analysis. Inference in this aim will be based on the estimation of effect sizes and 95% confidence intervals

Data Analysis

As a preliminary step, demographics, baseline measurements (weight, height, BMI), physical activity, diet, and psychosocial measures will be summarized across the aggregate sample and compared between groups using Analysis of Variance (ANOVA) for continuous variables, chi-squared analyses for categorical variables and non-parametric tests as appropriate. Any variables not balanced between randomization will be considered potential confounders in the subsequent models if these variables are correlated with the outcome under consideration (at a modest $p<.30$ level). The distribution of each of the primary and secondary study outcomes will be assessed using both parametric and graphical methods and transformed as necessary (e.g. Log transform towards normality). Potential distributions for the outcome variables include normal and binomial, and zero-inflated distributions. As a subsequent step, we will assess potential between-group differences in baseline variables between 'initial responders' and 'early non-responders' using the same approach as described above.

Primary Aim 1: We will compare treatment effects with respect to the primary outcome (% weight change from baseline) using a longitudinal mixed effects regression model. The model will simultaneously regress percent weight change at 4 and 12-month follow-up on treatment group, time, treatment x time and potential confounders of the treatment effect. Models will include a subject-specific intercept to adjust for repeated measurements within participant over time. Modeling is done using a likelihood-based approach and thus makes use of all available data without directly imputing missing outcomes, to produce consistent estimates of the regression parameters. Specification of contrasts for the treatment effect makes it possible to make all 2-way comparisons between treatment groups using a single model (e.g., Extended vs. Brief, Extended vs. Control, Brief vs. Control). Mixed models are flexible in that they allow for a wide range of specifications of the time effect (linear, non-linear) as well as distributions of the outcome (e.g., normal). Next, using a longitudinal regression model implemented with generalized estimating equations (GEE) with robust standard errors, we will examine treatment differences in clinically significant WL (≥5% WL)[105,106]. Specifically, we will regress indicators of clinically significant WL at 4 and 12-month follow-ups on treatment, time, treatment x time and potential covariates (e.g., variables not balanced by randomization) using binomial errors, a logit link function, and a working unstructured correlation to accommodate within-subject correlation. Finally, using a similar modeling approach to that above, we will examine treatment effects among the subset of early non-responders only.

Secondary Aim 1: We will estimate the incremental cost/kg WL in Brief and Extended, relative to Control. Costs will be compared using graphical methods, ANOVA and non-parametric methods as appropriate.

Secondary Aim 2: We will examine intervention effects on engagement (e.g., diet quality, physical activity, self-monitoring, video lessons viewed) and test whether engagement mediates effects of treatment on WL outcomes. First, using a series of mixed effects regression models, we will regress engagement over time on treatment group, time, treatment x time and potential confounders of the treatment effect. Models will be run separately for each of the engagement outcomes. Next. we will use a multiple mediation approach, in which all potential mediators (e.g., diet quality, physical activity, self-monitoring, video lessons viewed) are tested simultaneously using a product of coefficients method[107] with bootstrapped standard errors (5000 samples with replacement). We will estimate the path coefficients (*a* path: effects of treatment on changes in mediators over time and *b* path: effects of changes in the mediators on WL over time, controlling for baseline), as well as the indirect effect of treatment (*ab* path: effect of treatment on WL through the mediators). Models will be run separately for each of the primary and secondary WL outcomes.

It should be noted that Secondary Aims 1 and 2 will be analyzed first among the aggregate sample, and then among early non-responders only.

Secondary Aim 3: We will examine whether early non-responder status is a moderator of the treatment effect on WL outcomes using a similar analytic approach as that described for Primary Aim 1. Specifically, models for the primary and secondary WL outcomes will include main effects of treatment, time, potential moderator, as well as all two- and three-way interactions between these variables. Non-responder status will be considered a moderator if the treatment x moderator effect is significantly different than zero. Models for the primary WL outcome (percent weight loss) will be run using a mixed effects regression model and for the secondary weight outcome (% achieving a clinically significant WL) a GEE will be used. We will

examine the moderating role of non-responder status both continuously (% WL at 4 weeks) and categorically (e.g., <2% vs. 2-4%).

Exploratory Aim: Analysis will be conducted among early non-responders only. First, we will explore mechanisms through which PC may affect engagement and WL, including perceived support, self-efficacy and intrinsic/extrinsic motivation. First, we will consider potential mediators of PC effects on engagement, including self-efficacy and perceived support. We will use a similar approach to that described in Secondary Aim 3 in which a series of multiple mediators models implemented with a product of coefficients approach with bootstrapped standard errors (one for each engagement outcome) will be explored in order to generate effect estimates for the path coefficients (*a, b* and indirect effect *ab*) and associated 95% CI's. We will then explore whether these same constructs (support, self-efficacy and motivation) are potential mediators of WL outcomes. Our goal is not to conduct formal mediator models but rather to estimate effects to generate hypotheses in support of (or against) the conceptual model (Figure 1).

Next, we will explore additional potential moderators of the treatment effect (e.g. sex, race/ethnicity, self-efficacy, motivation) using a similar analytic approach as that described for Secondary Aim 2. Specifically, models for the primary and secondary WL outcomes will include main effects of treatment, time, potential moderator, as well as all two- and three-way interactions between these variables. A variable will be considered a moderator if the treatment x moderator effect is significantly different than zero. Models for the primary WL outcome (percent weight loss) will be run using a mixed effects regression model and for the secondary weight outcome (% achieving a clinically significant WL) a GEE will be used.

Missing data. Analyses will be on the intent-to-treat sample (everyone randomized will be included in the final analysis) under various assumptions about the missing data mechanism, apart from early non-responder analyses which will include only those participants who were able to be classified as initial responders or early non-responders at 4 weeks, which will not use an intent-to-treat approach (see Approach C.6). Sensitivity to these assumptions will be tested. Specifically, we will gather follow-up information and reasons for dropout regardless of protocol completion and censor at the point of loss. We will compare the robustness of our findings using two statistical approaches for handling missing data. First, we will use inverse probability weighting with propensity scores. This is a two-step method: 1) using logistic regression, the probability of missingness is modeled as a function of baseline covariates and baseline values of the outcome and 2) the inverse of the propensity scores (predicted probabilities of dropout from the first step) serve as weights in our regression model of the outcomes. Provided the data are missing at random (MAR) or that the probability of missingness can be fully explained by observable data, this approach produces asymptotically unbiased estimates. To allow for the possibility that the MAR assumption may not hold, we will also use a second approach, pattern mixture models, in which the distribution of the outcome is assumed to follow a mixture of two distributions: one for those who complete follow up and another for those who do not.

PROTECTION OF HUMAN SUBJECTS

1. Risks to Subjects

Human Subject Involvement and Characteristics: Participants in this study will be a sample of 450 men and women, aged 18-70 years, with a body mass index (BMI) of 25-45 kg/m^2, with regular Internet access. The lower age limit of 18 was selected to exclude children, given that children benefit from different types of dietary and physical activity interventions, particularly those with greater parental involvement. The upper age limit of 70 was chosen because older adults have more medical co-morbidities and may require more medical supervision. Given that weight loss (WL) is recommended for those who are overweight or obese, 25 kg/m^2 was selected as the lower end of the BMI range since this is the threshold used to classify overweight. An upper BMI range of 45 kg/m^2 was included, as individuals with a BMI >45 have additional medical co-morbidities and may require more intensive weight loss programs or bariatric surgery. Participants must have regular (i.e., daily) Internet access (defined as owning a smartphone with Internet capabilities, and/or having a broadband or wi-fi connection at home or work) to be eligible for this study. However, according to recent Pew Research data, 88% of Americans use the Internet and this percentage is upwards of 98% among individuals ≤49 years of age. Further, Internet usage was found to be similar across racial categories (Caucasian: 88%,

Hispanic 88%, Black 85%) and 73% of adults have high-speed broadband service at home. Therefore, it is likely that very few individuals will be excluded from this study due to a lack of regular Internet access.

Listed below are additional exclusionary criteria. These inclusion/exclusion criteria were developed to ensure participant safety and limit the number of individuals who would be excluded for obesity-related medical conditions or comorbidities. Given that dissemination of this program is a broader goal of this study, we also wanted to ensure that the study sample was more inclusive and representative of the overweight and obese population. To ensure safety, individuals with a history of heart disease, diabetes, or cancer, will be required to provide physician consent prior to enrolling.

Exclusionary criteria:

1) Currently pregnant, <6 months post-partum, nursing, or planning to become pregnant within the next 12 months.
2) Current enrollment in a commercial WL program or other WL research study
3) Currently taking WL medications or previously had bariatric surgery
4) Inability to walk 2 blocks without stopping
5) Any medical condition for which WL, dietary restriction, or physical activity is contraindicated

Sources of Research Material: Data will be obtained specifically for research purposes via direct measurement and questionnaires. Height and weight will be directly measured, physical activity will be objectively-assessed using the Actigraph GT9X Link accelerometer, and demographic information, health history, and dietary and weight control practices will be assessed via questionnaire. Further, intervention adherence data, the number of phone coaching calls completed, the length of each phone call, and interventionist time will be obtained directly from the intervention platform and database.

Potential Risks: The risks associated with this study are quite minimal. The intervention instructs participants to engage in moderate-intensity exercise which gradually progresses to 250 minutes/week. Although safety precautions will be taken (e.g., gradual exercise progression, information on exercise safety is provided), it is possible that an individual could experience some muscle soreness or an injury as a result of the exercise. Further, the risk of a sudden cardiac event occurring during exercise is very minimal and is estimated to be approximately 1 per every 36.5 million hours of moderate-to-vigorous exertion. In addition, participants will be instructed to reduce calorie intake by 500 to 1000 calories per day. It is possible that participants may engage in unhealthy dietary practices, but this is not likely and precautions will be taken to minimize this risk. Finally, there is the risk that the participant will not lose weight as a result of this program. However, this is a potential risk for any weight loss program or treatment.

2. Adequacy of Protection Against Risks

Recruitment and Informed Consent: Participants will be recruited through advertisements in local newspapers, direct mailings, Internet postings, and through business and community contacts. Interested individuals will respond to study advertisements by calling the Weight Control and Diabetes Research Center (WCDRC) or by going to the study website. An overview of the study will be described either via text on the study website, or verbally via the phone and interested individuals will be screened for eligibility. Eligible participants will then be scheduled for an orientation session either in-person, via phone or video conferencing where the study will be explained in greater detail. Individuals interested in the study will sign the informed consent document approved by the Miriam Hospital's Institutional Review Board either in-person or electronically via Redcap .

Protection Against Risk: To minimize risk, participants will be initially screened for any health conditions that would limit participation in a weight loss program and physician consent will be required for individuals with a history of diabetes, cancer, or heart disease. Further, as part of the initial intervention session, participants will be advised about safe weight loss practices. A rate of weight loss of 1-2 pounds per week is recommended and has been shown in other studies to be a safe rate of weight loss. Moreover, participants will have their

weight objectively-assessed at baseline, 4, and 12 month assessment visits. If the rate of WL is too severe, or if the participant reports unsafe weight control practices at these visits, the Principal Investigator will discuss these issues with the participant. Further, as a safety precaution, gradual progression of exercise will be prescribed within the intervention. Finally, protection against the risk of a breach of confidentiality will be minimized through careful adherence to best practices for data collection and management. Dr. Unick and staff at the WCDRC have extensive history conducting clinical trials and management of identifiable data from participants randomized to a weight management program.

3. Potential Benefits of the Proposed Research to Subjects and Others

Participants will receive a behavioral weight loss intervention which teaches healthy eating and exercise behaviors. This has the potential to result in clinically significant weight losses and improvements in various health outcomes. Thus, while the potential risks of this study are quite minimal, the potential benefits are quite significant.

4. Importance of Knowledge to be Gained

Effective weight loss programs are critical for combatting the current obesity epidemic. This study is significant because it seeks to develop and evaluate a weight loss program, tailored to non-responders, which can be delivered entirely remotely; thus having great dissemination potential. Findings for this study could have important implications for obesity treatment.

5. Educational Training of Staff

All investigators and staff will be required to obtain education regarding the protection of volunteers in research from their own institution. The Miriam Hospital follows this plan:
In June, 2005, the Office of Research Administration contracted with CITI, a Collaborative Institutional (modular) Training Initiative program, for our Human Subjects protection and HIPAA training for all research personnel. Currently this program offers our researchers a basic human subject's protection course as well as a refresher course which we require every three years. Documentation of successful completion is automatically generated and should be printed directly by the researcher. For further information regarding Lifespan's Human Subject's Protection: http://www.lifespan.org/research/IRB/MandatoryEdguidance.asp
Additional and continuing education opportunities for clinical researchers include the Office of Research Administration newsletter that is circulated to > 900 recipients every 6 weeks. Relevant information concerning research review is available on the ORA web page at **www2.lifespan.org/research/**. In addition to standard institutional research information, the web page contains links to other sites such as CenterWatch, NIH, PRIM&R/ARENA.

DATA SAFETY MONITORING PLAN

The purpose of this study is to examine whether the addition of phone coaching (PC) for early non-responders enrolled in an Internet-delivered weight loss (IDWL) program improves treatment outcomes. Participants in this study will be asked to decrease their calorie intake and increase their physical activity to reduce body weight. Healthy weight loss practices will be recommended using previously tested diet and exercise prescriptions which have been shown to be safe. Thus, the focus of the Data Safety Monitoring Plan (DSMP) will be on monitoring the progress of the study (this includes recruitment, data collection, data management/quality control, adverse events, etc.) and reporting any concerns to the IRB and NIH study sponsor. The timing, frequency of reporting, and frequency of review are outlined below.

Monitoring	Reporting Frequency	Frequency of Review
Subject Enrollment	Monthly by Dr. Unick	Bi-annually to Independent Safety Officer
IRB Compliance	Annually by Dr. Unick	Annually to Independent Safety Officer

		and NIH
Adverse Events	Continuously (see below)	Bi-annually to Independent Safety Officer and annually to IRB and NIH
Quality Control	As needed by Dr. Unick	Annually to NIH

Independent Safety Officer

Dr. Amy Gorin has agreed to serve as the Safety Officer for this trial. She is a Professor in the Department of Psychological Sciences at the University of Connecticut and is nationally recognized for her work in the area of behavioral weight control. She has been the PI or Co-I on numerous NIH-funded grants; thus Dr. Gorin is well qualified to monitor the progress and safety of this trial.

As indicated in the above table, our proposed DSMP will require frequent reporting to the Safety Officer and NIH study sponsor, and both parties will review and approve this protocol prior to the start of the trial. Dr. Gorin will be provided with study progress reports in 6 month intervals. These reports will include information on recruitment, rate of subject enrollment, efforts to recruit minority participants, participant retention, demographic characteristics of study participants, reasons for dropout, and adverse events. After reviewing these data, Dr. Gorin will indicate to Dr. Unick and NIH whether the study is progressing appropriately or whether changes are needed. At any point during the trial the Safety Officer can recommend that a phone call be held to discuss any issues with the progress or safety of the trial.

Adverse Events and Serious Adverse Events

An adverse event is defined as any untoward or unfavorable medical occurrence in a human subject, including any abnormal sign (for example, abnormal physical exam or laboratory finding), symptom, or disease, temporally associated with the subject's participation in the research, whether or not considered related to the subject's participation in the research. At each assessment visit, participants will be queried regarding any adverse events using an Adverse Events Form. In addition, information about adverse events may be reported by the participant on a coaching call. All interventionists will be trained to identify adverse events and to report any AEs to Dr. Unick using an Interim Adverse Event Form. All AEs will be reported to the IRB, NIH, and Safety Officer as indicated above. The IRB at The Miriam Hospital is a fully authorized Institutional Review Board that provides oversight to research conducted at The Miriam Hospital, Rhode Island Hospital, and affiliated Brown Medical School. It functions in compliance with the congressional statutes governing Assurance of Compliance with Health and Human Services (HHS) Regulations for Protection of Human Research Subjects. This board will be providing oversight to the proposed study.

Serious adverse events (SAEs) are defined as any adverse event that meets any of the following criteria: fatal or life-threatening, result in significant or persistent disability, require or prolong hospitalization, result in a congenital anomaly/birth defect, or are important medical events that investigators judge to represent significant hazards or harm to research subjects. Any adverse event that meets any of these criteria will be documented as an SAE and an SAE form will be completed by the study investigators. Serious adverse events will be reported to the NIH and The Miriam Hospital's IRB within 24 hours of knowledge of the event.

Participant Confidentiality. Participant confidentiality is of utmost importance for this study and guidelines outlined by our IRB will be followed. Participant data confidentiality will be protected through a multi-tiered approach including data collection, data handling, and data distribution processes to ensure anonymity both during and after the study. Participant information collected by the research staff will contain only a non-identifiable study ID. A separate form linking study ID and participant identifiers (name, address, contact names and addresses) will be maintained in a locked file.

Quality Control. All staff involved in data collection will be trained by the investigators and must demonstrate competence in administering all measures. The research assistant will review all assessment data for accuracy and completion. The investigators will conduct error checking and preliminary analyses of all data to

ensure accuracy. Hard copies of data will be stored in a locked filing cabinet and electronic data file will be password protected and backed up.
