# Supplementary material for: An Adaptive Telephone Coaching Intervention for Patients in an Online Weight Loss Program: A Randomized Clinical Trial
Source: JAMA Netw Open. 2024 Jun 7;7(6):e2414587. doi: 10.1001/jamanetworkopen.2024.14587 (PMC11161849; doi:10.1001/jamanetworkopen.2024.14587)
Supplement: Supplement 2. — eMethods. Description of Cost Analyses eTable. Summary of Coaching Costs eFigure 1. Examination of Percent Weight Change at 4 and 12 Months Among Early Suboptimal Responders Only (n=346) eFigure 2. Examination of 1-Month Weight Loss as a Treatment Moderator of Weight Loss at (a) 4 Months and (b) 12 Months by Randomization Assignment [file jamanetwopen-e2414587-s002.pdf]

Supplemental Online Content

Unick JL, Pellegrini CA, Dunisger SI, et al. An adaptive telephone coaching intervention for patients in an online weight loss program. *JAMA Netw*. *Open*. 2024;7(6):e2414587. doi:10.1001/jamanetworkopen.2024.14587

eMethods. Description of Cost Analyses

eTable. Summary of Coaching Costs

eFigure 1. Examination of Percent Weight Change at 4 and 12 Months Among Early Suboptimal Responders Only (n=346)

eFigure 2. Examination of 1-Month Weight Loss as a Treatment Moderator of Weight Loss at (a) 4 Months and (b) 12 Months by Randomization Assignment

This supplemental material has been provided by the authors to give readers additional information about their work.

© 2024 Unick JL et al. *JAMA Network Open.*

eMethods. Description of Cost Analyses

A secondary aim of this study was to assess the cost-effectiveness of the supplemental phone coaching for early non-responders when added to a pre-existing Internet-based WL program. Costs were estimated from a payer perspective, reflecting the costs that a healthcare system would need to pay to provide supplemental coaching to early non-responders enrolled within Internet-based WL treatment. No costs were attributed to the Internet program given that it was already developed, completely automated, and provided to everyone, regardless of whether they received coaching (i.e., the cost of CONTROL was considered to be $0 since no coaching was provided). Costs associated with coaching included interventionists' time, office space, access to telephone service, and a one-time cost to train coaches. Interventionist time was valued at the median wage using payroll records ($33.67/hour + 24.3% fringe benefits) and included the time spent on coaching calls (i.e., call time) as well as non-call time (e.g., reviewing self-monitoring data, emailing meal plans [week 5 only], rescheduling calls). Coaches tracked the amount of time spent on each participant. Office space was calculated using the mean rent in Providence RI ($3569/year for 150 square foot office) and annual phone service cost was $540 per line. Although all coaching for this study required less than one interventionist at full-time effort, office space and phone line service would be required for a full year to run this type of program annually. Therefore, the yearly cost for office space and phone service was divided between BRIEF (38.0%) and EXTENDED (62.0% - percentages calculated using ratio of interventionist time per BRIEF / EXTENDED participant). A one-time, 10-hour upfront interventionist training cost was also included for each condition. Costs associated with the research study (e.g., time spent logging research data, study-related meetings, recruitment of participants, development of coaching intervention) were not included. The cost per enrolled participant was calculated by summing all coaching costs within treatment arm and dividing by the number of participants enrolled in that treatment arm. We elected to examine the cost per enrolled participant, rather than the cost per participant who received coaching, because if this program were to be implemented in the real world, from a budgetary planning perspective, it would be more helpful to know the upfront cost per enrolled participant prior to initiating the program. The incremental cost-effectiveness ratio was also calculated and defined as the difference in cost between a coaching intervention and CONTROL, divided by the difference in their effect on WL (see main manuscript for these data).

Over the course of the trial, n=111 BRIEF participants (out of n=150 randomized) and n=119 EXTENDED participants (out of 150 randomized) were prescribed coaching. Total interventionist time spent across all BRIEF participants was 188.7 hours (time on calls: 139.3 hours, non-call time: 49.4 hours). Total interventionist time spent across all EXTENDED participants was 531.9 hours (time on calls: 400.0 hours, non-call time: 131.9 hours). A breakdown of total costs is shown in **eTable 1.**

eTable. Summary of Coaching Costs

	Total cost across all participants		Cost per enrolled participant	
**Total intervention costs***	**Brief**	**Extended**	**Brief**	**Extended**
Interventionist time - on calls	$5,830.17	$16,742.33	$38.87	$111.62
Interventionist time - not on calls	$2,067.27	$5,519.90	$13.78	$36.80
Office space cost (assumes one office)	$1,356.22	$2,194.18	$9.04	$14.63
Phone usage (assumes one phone line)	$171.00	$279.00	$1.14	$1.86
One-time interventionist training (10 hours)	$418.52	$418.52	$2.79	$2.79
**Total cost**	$9,843.17	$25,153.93	$65.62	$167.69

* Assumes one interventionist at full-time effort

© 2024 Unick JL et al. *JAMA Network Open.*

eFigure 1. Examination of Percent Weight Change at 4 and 12 Months Among Early Suboptimal Responders Only (n=346)

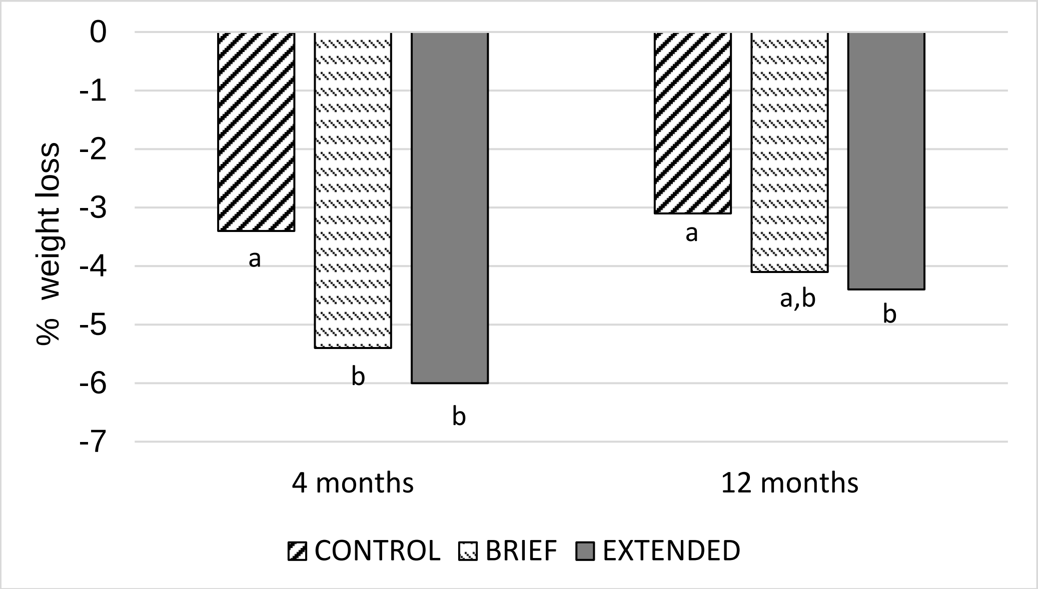

Values with different superscripts are significantly different from one another ($p<0.05$)

© 2024 Unick JL et al. *JAMA Network Open.*

eFigure 2. Examination of 1-Month Weight Loss as a Treatment Moderator of Weight Loss at (a) 4 Months and (b) 12 Months by Randomization Assignment

a)

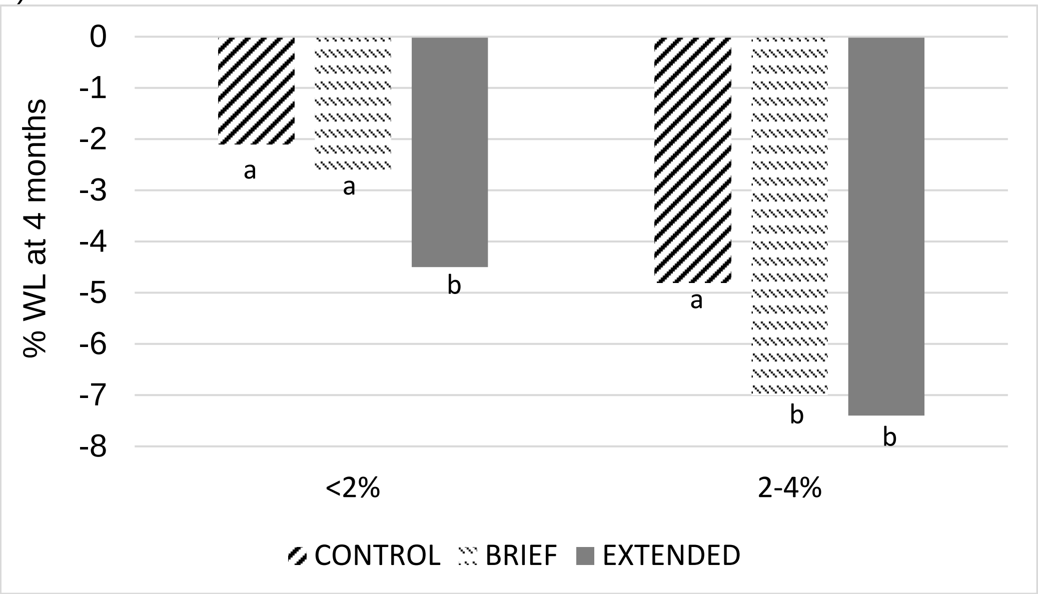

b)

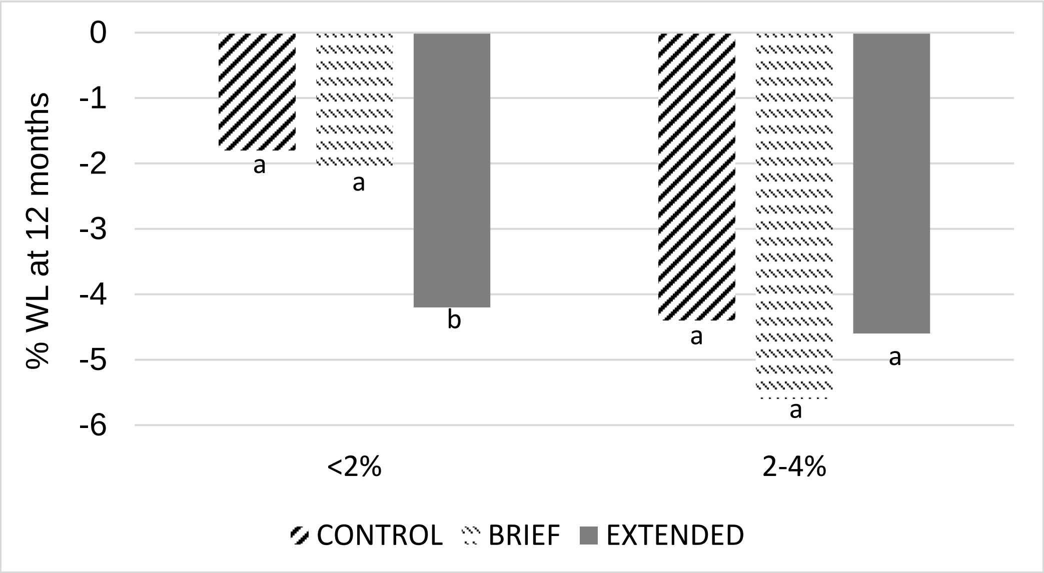

Within 1-month WL categories, values with different superscripts are significantly different from one another ($p<0.05$)

© 2024 Unick JL et al. *JAMA Network Open.*
