# Supplementary material for: An Adaptive Telephone Coaching Intervention for Patients in an Online Weight Loss Program: A Randomized Clinical Trial
Source: JAMA Netw Open. 2024 Jun 7;7(6):e2414587. doi: 10.1001/jamanetworkopen.2024.14587 (PMC11161849; doi:10.1001/jamanetworkopen.2024.14587)
Supplement: Supplement 3. — Data Sharing Statement [file jamanetwopen-e2414587-s003.pdf]

# Data Sharing Statement

Unick. Efficacy of an Adaptive Intervention for Patients in an Online Weight Loss Program. *JAMA Netw Open*. Published June 07, 2024. doi:10.1001/jamanetworkopen.2024.14587

**Data**
**Data available:** Yes
**Data types:** Deidentified participant data
**How to access data:** Deidentified data is available upon request to Jessica Unick (junick@lifespan.org)
**When available:** beginning date: 08-30-2024

**Supporting Documents**
**Document types:** None

**Additional Information**
**Who can access the data:** N/A
**Types of analyses:** N/A
**Mechanisms of data availability:** N/A
